# M1-like macrophage polarization prevails in young children with classic Hodgkin Lymphoma from Argentina

**DOI:** 10.1038/s41598-019-49015-1

**Published:** 2019-09-03

**Authors:** O. Jimenez, M. H. Barros, E. De Matteo, M. Garcia Lombardi, M. V. Preciado, G. Niedobitek, P. Chabay

**Affiliations:** 1 Multidisciplinary Institute for Investigation in Pediatric Pathologies (IMIPP), CONICET-GCBA, Molecular Biology Laboratory, Pathology Division, Ricardo Gutiérrez Children’s Hospital, Buenos Aires, Argentina; 2 Institute for Pathology, Unfallkrankenhaus Berlin, Berlin, Germany; 3 Oncology Division, Ricardo Gutiérrez Children’s Hospital, Buenos Aires, Argentina

**Keywords:** Immunopathogenesis, Infection

## Abstract

The microenvironment in classical Hodgkin lymphoma (cHL) comprises a mixture of different types of cells, which are responsible for lymphoma pathogenesis and progression. Even though microenvironment composition in adult cHL has been largely studied, only few groups studied pediatric cHL, in which both Epstein Barr virus (EBV) infection and age may display a role in their pathogenesis. Furthermore, our group described that EBV is significantly associated with cHL in Argentina in patients under the age of 10 years old. For that reason, our aim was to describe the microenvironment composition in 46 pediatric cHL patients. M1-like polarization status prevailed in the whole series independently of EBV association. On the other hand, in children older than 10 years, a tolerogenic environment illustrated by higher FOXP3 expression was proved, accompanied by a macrophage polarization status towards M2. In contrast, in children younger than 10 years, M1-like was prevalent, along with an increase in cytotoxic GrB+ cells. This study supports the notion that pediatric cHL exhibits a particular tumor microenvironment composition.

## Introduction

The interaction between tumor and its microenvironment has been a key topic in cancer research for the last years. cHL has a unique morphological presentation with a minority of neoplastic cells, and a large majority of non-malignant reactive immune cells^1,2^. This extensive milieu of non-malignant cells is recruited by Hodgkin Reed–Sternberg (HRS) tumor cells and comprises B and T cells,together with eosinophils, neutrophils, mast cells, fibroblasts and macrophages^1–3^. HRS cells secrete cytokines, chemokines, and other immunomodulatory factors, such as IL-10, CCL17, galectin-1, and indoleamine 2,3-dioxygenase (IDO) that both recruit Th2 and regulatory FOXP3+ CD4+ T cells (Treg), favoring the differentiation of tumor-infiltrating T cells into regulatory and Th2 cells. HRS cells also secrete macrophage migration inhibition factor, which induces macrophage M2 polarization, also rendering a tumor microenvironment favorable for tumor cell growth and survival^4^.

Most studies about tumor microenvironment composition in cHL were performed in adults and a minority in pediatric cases. Moreover, these pediatric studies still wait for further validation^5–7^. This is a crucial point since the immune response against HRS cells and consequently the tumor microenvironment composition may differ between adult and children^6,8,9^. For instance, EBV+ pediatric cHL cases display predominantly GrB+ cytotoxic/Th1 microenvironment profile, along with an increase in CD68+ and CD163+ cells and M1-like polarized macrophages^6,8,9^.

The evaluation of macrophage polarization on tissue sections is challenging. We published previously an immunohistochemical-based approach to evaluate this polarization^10^. This was based in experimental results, showing that STAT1 expression was proved in M1 macrophages in response to types I, II or III interferon, whereas its phosphorylated form (pSTAT1) binds to the promoter region of interferon-stimulated genes^11–14^; on the other hand, CMAF is expressed in M2 macrophages that produce interleukin (IL)-10, given that is an important transcription factor for IL-10 production^15–17^.

We performed this study in order to study microenvironment composition in pediatric cHL, including an independent and geographically different series of cases from a single institution, in a region where EBV association is significant in patients under 10 years of age, and cHL subtype distribution is different from previous studies^18^.

## Results

Forty six pediatric cHL patients were included in this analysis. The mean age of the children was 9 years (3 to 16 years) and 76.1% (35/46) of cases were EBV-associated. Mixed cellularity (MC) was the most prevalent subtype (27/46 cases, 58.7%), and was significantly associated to the <10 years age group (p = 0.0004, Fisher exact test). Eighty percent (28/35) of EBV-associated cases were in younger children (p = 0.002, Fisher’s exact test). EBV presence was statistically related to the MC subtype (25/35 EBV+ cases, 71.4%) over the nodular sclerosis (NS) subtype (7/35 EBV+ cases, 20%) (p = 0.0085, Fisher exact test) (Table S1).

Comparable numbers of CD4+, CD8+, FOXP3+ and GrB+ cells were observed at the tumor microenvironment (Table 1) in the 46 patients series. A statistically significant positive correlation was found only between FOXP3+ and GrB+ cells (r = 0.30, p = 0.03, Spearman correlation test) (Table S2). Young children showed a tendency of higher numbers of CD4+ and CD8+ cells/mm^2^ (p = 0.08 and p = 0.089, respectively, Mann-Whitney test) and were associated with lower FOXP3+ cells/mm^2^ cell count (p = 0.017, Mann-Whitney test), in comparison with old children (Table 1). No significant differences were found among the cellular markers investigated between EBV+ and EBV− cases (p > 0.05, Mann Whitney test). Detailed description of 46 cHL patients is provided in the Table 1 and Supplemental Tables.

**Table 1:** Description of analyzed cell subsets according to age-group and EBV-association in pediatric classical Hodgkin lymphoma.

Cell Subset	Age group			EBV status		
	≤10 y	>10 y	p	Positive	Negative	p
Mean CD4+ cells/mm^2^	766	733	0.08	771	750	0.67
Mean CD8+ cells/mm^2^	773	572	0.089	707	592	0.26
Mean FOXP3 cells/mm^2^	1008	1459	0.017	1047	1405	0.32
Mean GrB+ cells/mm^2^	613	556	0.3	528	736	0.91
Mean CD68+ pSTAT1+ cells/mm^2^	247	130	0.021	215	178	0.69
Mean CD68+ CMAF+ cells/mm^2^	20	69	0.09	33	49	0.29
Mean CD163+ pSTAT1+ cells/mm^2^	165	125	0.56	153	147	0.66
Mean CD163+ CMAF+ cells/mm^2^	67	121	0.017	81	102	0.08

GrB: Granzyme B. EBV: Epstein-Barr virus. Y: year. P-values from Mann-Whitney test.

**Figure 1:**
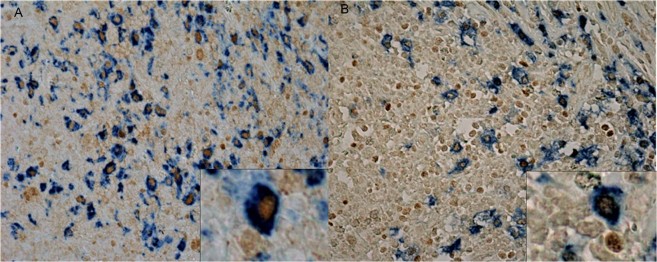
Representative double immunohistochemical staining undertaken on sections from cHL biopsies. Cytoplasmic CD68+ staining combined with nuclear pSTAT1+ macrophages (**A**). Cytoplasmic CD163+ staining combined with nuclear CMAF+ macrophages (**B**). Original magnification: ×400.

CD68+ pSTAT1+ macrophages prevailed in relation to CD68+ CMAF+ macrophages (mean 197 cells/mm^2^ and 47 cells/mm^2^, respectively, p < 0.0001, Mann Whitney test), as well as CD163+ pSTAT1+ macrophages in relation to CD163+ CMAF+ macrophages (mean 131 cells/mm^2^ and 77 cells/mm^2^, respectively, p = 0.006, Mann Whitney test) (Fig. 1). Predictably, a significant correlation was found between CD68+ pSTAT1+ and CD163+ pSTAT1+ (p < 0.0001, Spearman correlation test), and between CD68+ CMaf+ and CD163+ CMaf+ cells (p < 0.0001, Spearman correlation test). Young children exhibited increased numbers of CD68+ pSTAT1+ macrophages/mm^2^ (p = 0.021, Mann-Whitney test), while older children showed higher numbers of CD68+ CMAF+ and CD163+ CMAF+ macrophages/mm^2^ (p = 0.09 and p = 0.017, respectively) (Table 1). EBV presence did not showed any influence on macrophage composition (Table 1).

A positive correlation was proved between CD68+ pSTAT1+ macrophages and GrB+ cells (r = 0.46, p = 0.002, Spearman’s correlation), while CD68+ CMAF+ macrophages exhibited an inverse correlation with CD8+ cells (r = −0.49, p = 0.001, Spearman’s correlation). Regarding CD163, only an inverse correlation was found among CD163+ CMAF+ macrophages and CD8+ cells (r = −0.32, p = 0.049, Spearman’s correlation) (Table S2).

Regarding macrophage polarization and considering CD68 as macrophage marker, 76.9% (30/39) of cases displayed M1-like polarization, 15.4% (6/39) M2-like polarization and 7.7% (3/39) comparable amount of M1- and M2-like macrophages. When CD163 was used as macrophage marker, 54.5% (18/33) of cases turned out to be M1-like polarized, 33.3% (11/33) M2-like polarized and 12.1% (4/33) exhibited equivalent numbers of M1- and M2-like macrophages.

M1-like polarization was statistically prevalent in patients younger than 10 years old, while, in contrast, M2 polarized cells presence were associated to older patients, considering both CD68 and CD163 as macrophage markers (p = 0.006 and p = 0.0015, respectively, Fisher exact test) (Table S3). Regarding EBV-association, no differences were observed when CD68 was considered as macrophage marker. However using CD163, EBV-associated cases displayed frequently M1-like polarization (66.7% of cases, 16/24), while 66.7% (6/9) of EBV-negative cases exhibited M2-like polarization (P = 0.032, Fisher’s exact test) (Table S3).

Given that older children showed an immunoregulatory-rich tumor microenvironment, we evaluated if this characteristic could affect the macrophage composition. For this purpose, a ratio of FOXP3/GrB >1.5 as indicative of an immunoregulatory-rich microenvironment was defined, as published previously^8^. An immunoregulatory-rich tumor microenvironment was associated with lower mean of CD68+ pSTAT1+ cell numbers (mean 163 cells/mm^2^ for FOXP3+/GrB ratio >1.5 vs. mean 270 cells/mm^2^ for FOXP3+/GrB ratio ratio <1.5, p = 0.028, Mann-Whitney test). In fact, M1-like polarization was the exclusive pattern observed in patients with FOXP3/GrB ratio <1.5 defined by CD68+ pSTAT1+ approach. No difference was highlighted when CD163 was considered.

**Figure 2:**
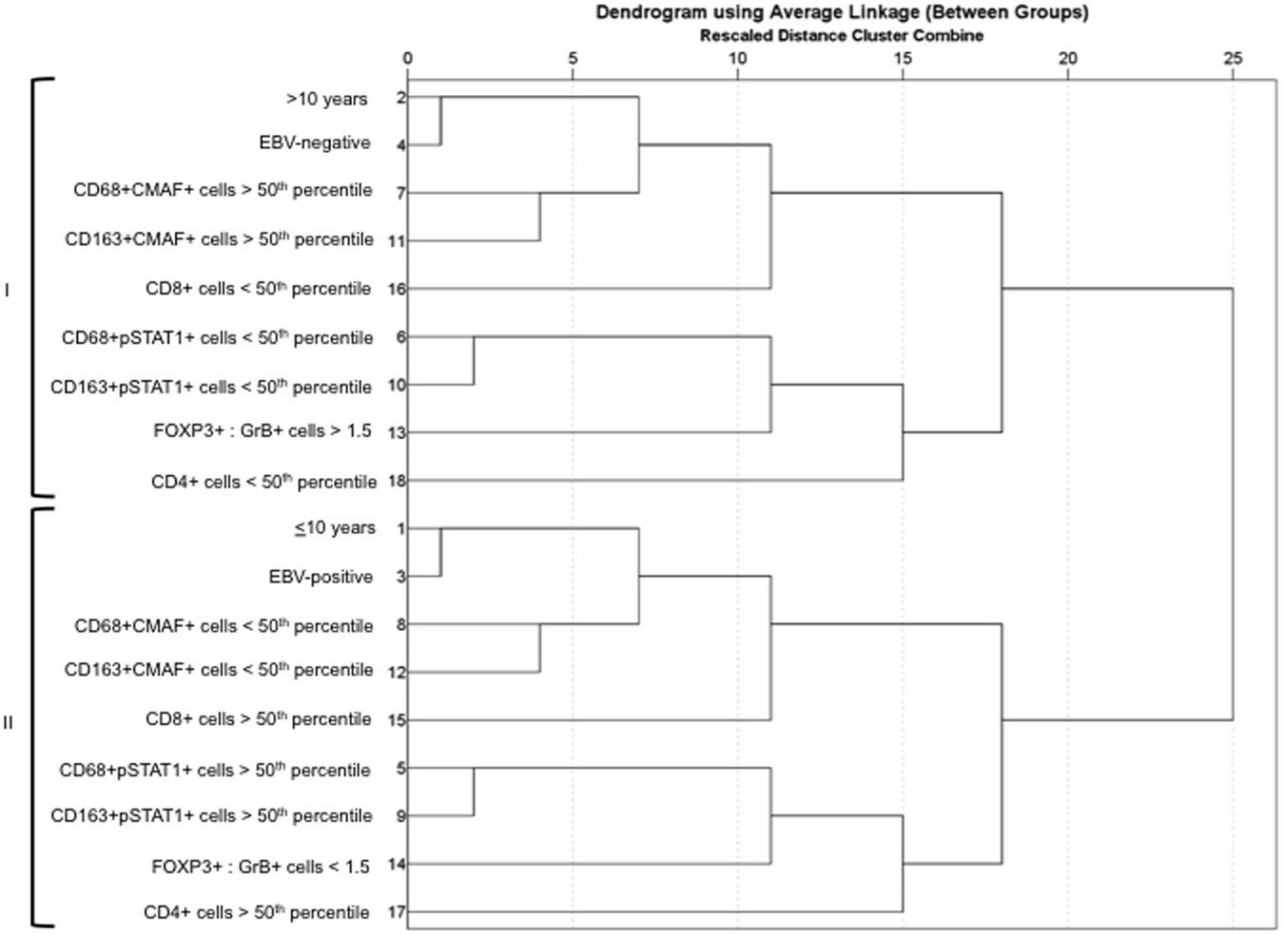
Dendrogram using average linkage obtained by hierarchical cluster analysis. Two main identified clusters (I and II) are indentified by brackets. Num, order of variable input. EBV: Epstein-Barr virus. GrB: Granzyme B.

Finally, hierarchical cluster analysis was conducted to identify underlying patterns of tumor microenvironment cell subsets, in relation to both EBV status and age group (Fig. 2). In this analysis, 2 distinctive clusters were observed: cluster I included the EBV− negative cases, those with high M2-like macrophages cell count, as demonstrated by CD68+ CMAF+ and CD163+ CMAF+ above the 50th percentile, and predominance of FOXP3+ over GrB+ cells, given that the ratio Foxp3+: GrB+ >1.5 prevails in this cluster. In contrast, the cluster II is mainly formed by EBV-associated cases and those with increased numbers of M1-like macrophages, confirmed by CD68+ pSTAT1+ and CD163+ pSTAT1+ cases above the 50th percentile, and predominance of GrB+ over FOXP3+ cells, since the ratio GrB+: Foxp3+ is higher than 1.5.

## Discussion

In pediatric cHL, only two studies were performed in children from developed socioeconomic populations^5,7^, while one group has extensively studied a pediatric population from a developing country, Brazil^6,8,9^. Gupta *et al*.^5^ and Englund *et al*.^7^ performed tumor microenvironment analysis in pediatric cHL cases from developed population (Canada and Sweden, respectively), in which cHL incidence, rate of EBV-association and subtype distribution is different. In developed countries, nodular sclerosis (NS) subtype is prevalent over mixed cellularity (MC). In fact, NS subtype was described in 91.6% of cases demonstrated by Gupta *et al*.^5^, and in 69% of cases reported by Englund *et al*.^7^. In line with this, Barros *et al*.^6,8,9^ described in their pediatric series that 69% of cases were NS subtype, even though their study was performed in a developing population. In contrast, in our study 58.7% of cases were MC subtype. EBV was associated with 25% of pediatric cases from a developed population published Englund et al., particularly in patients younger than 10 years old^7^. Meanwhile 44.8% of cases were EBV-associated by Barros *et al*. in pediatric cHL cases from a developing country, without correlation with age-groups^6,8,9^. In contrast, we observed an increase in EBV-association in our series from a developing population along with a correlation with young age-group.

Barros *et al*. suggested a shift from a cytotoxic to a regulatory profile in the tumor microenvironment of pediatric cHL with the increase of the age^8^. In line with this, we observed a regulatory profile in children older than 10 years old, confirmed by a higher FOXP3+ cell counts. Nevertheless, an increment on the numbers of GrB+ cells was not observed in younger patients, in spite of EBV prevalence on this specific group, which would be expected to trigger an antiviral cytotoxic profile. However it is remarkable that EBV-associated cases exhibited higher means of CD8+ cells/mm^2^, as described previously^8^. The reasons why older children develop a tumor microenvironment rich in Treg cells, similar to adult cases^19^, are not clear yet. One possible explanation could be the increase of Treg cells numbers and suppressive function with the aging of the immune system^20^.

Polarized M1 macrophages are generally pro-inflammatory and anti-tumoral. In contrast, M2 macrophages were described as an immunoregulatory phenotype, which mediate tumor promotion^21^. In cHL, the immunomodulatory phenotype adopted by macrophages may be the potential mechanism by mean of which they promote immune evasion^22^. In this study we observed an increase in M1-like macrophages cell count and M1-like polarized tumor microenvironment in younger children. Also, this kind of polarization was linked to cytotoxic tissue signature, as evidenced by the increase in GrB+ cells in M1-like polarized cases. In contrast, higher numbers of M2-like macrophages and M2-like polarized tumor microenvironment was observed in older patients and related to an immunoregulatory-rich environment, in line with previous results^9^. Together, our results support previous hypothesis that the tumor microenvironment in pediatric cHL is influenced by age, and a possible cross-talk between cells of adaptive and innate immune system in this microenvironment may contribute to the macrophage polarization^6,8,9^.

In summary, a predominance of M1-like polarized macrophages in young children, as well as M2-like polarized macrophages and Treg cells in old children was observed, supporting the notion that pediatric cHL exhibits a particular tumor microenvironment composition when compared with adult cases^6,8,9,23–25^.

## Methods

### Patients and samples

Formalin-fixed paraffin-embedded (FFPE) biopsy samples from 46 patients were collected retrospectively, on the basis of the availability of sufficient material, from the archives at Pathology Division, Ricardo Gutierrez Children’s Hospital in Buenos Aires, Argentina, from 1990 through 2012, and a tissue microarray (TMA) was constructed as described^26^. From each case, two 3-mm-diameter cores, selected from two different representative tumor areas rich in HRS cells selected by the pathologists EDM and MHB, were included^26^.

The Ricardo Gutierrez Children Hospital Ethics Committee on Research (CEI) approved the study, and the subjects gave informed consent to the study. Diagnosis was revised according to the updated WHO scheme for lymphomas^27^. All methods were performed in accordance with the relevant guidelines and regulations.

### Immunohistochemistry

Tissue Microarray (TMA) blocks including 46 cHL cases were constructed, as previously described^6,8,9^. For each patient, two 1-mm-diameter cores, selected from two different representative tumor areas rich in Hodgkin and Reed-Sternberg cells, were included, as described^6,8,9^. All cases showed cores with representative tumor microenvironment. Immunohistochemistry (IHC) staining was performed on TMA sections with antibodies: CD4 (Leica, Newcastle, UK), CD8 (Dako, Gloustrup, Denmark), FOXP3 (Treg) (Abcam, Cambridge, UK), Granzyme B (GrB) (AbD Serotec, Oxford, UK). After incubation with primary antibody, immunodetection was performed using ZytoChem Plus HRP polymer kit (Zytomed Systems, Berlin, Germany), employing diaminobenzidine (DAB) chromogen as substrate, as described^6,8,9^. Sections were counterstained with hematoxylin.

In addition, macrophage polarization was assessed by double immunohistochemistry, as described previously^10^. pSTAT1 or CMAF (Santa Cruz Biotechnology, Dallas, USA) were used as first primary antibodies and the detection of bound antibodies was performed using ZytoChem Plus HRP polymer kit (Zytomed Systems, Berlin, Germany), employing diaminobenzidine (DAB) as chromogen^10^. Then, CD68 (Dako) or CD163 (Novocastra, Wetzlar, Germany) antibodies were incubated as second antibodies, followed by detection with AP Polymer System (Zytomed Systems, Berlin, Germany), wiht Blue Alkaline Phosphatase substrate kit (Vector Laboratories, Burlingame, USA) as substrate. The sections were not counterstained^10^. Co-expression of pSTAT1 together with CD68 or CD163 was used to identify M1-polarized macrophages, while co-expression of CMAF in conjunction with CD68 or CD163, was performed to identify M2-polarized macrophages^20^. The ratio M1: M2 per case was assessed for each marker combination, and a polarized response (M1 > M2 or M2 > M1) was defined as one cell population 1.5x higher than the other^10^.

### Tumor microenvironment analyzes

For lymphocyte and macrophage markers evaluation, the slides were scanned with Aperio LV1 digital pathology slide scanner (Leica Biosystems, CA, USA) at a 200× magnification. Using Aperio Image Scope software (Leica Biosystems, CA, USA), each core was extracted as separated JPG file. After, the numbers of labeled lymphocytes were determined per 1 mm^2^ using the image analysis free software Image J^28^. The cells were counted optically without the use of plug-in.

### Epstein-barr virus association

Epstein-Barr virus association was evaluated by EBERs *in situ* hybridization (EBER-ISH) and immunohistochemistry. EBER-ISH was performed on FFPE tissue sections using fluorescein isothiocyanate (FITC)-conjugated EBERs oligonucleotides as probes, following the manufacturer’s instructions (Dako). A monoclonal antibody anti-FITC labeled with alkaline phosphatase was used for the detection of EBERs probes (Dako), as described^29^.

Immunostaining was used to localize viral LMP1 expression in tumor cells, using monoclonal antibodies CS1-4 (Dako). IHC detection of primary antibody was carried out using a universal streptavidin–biotin complex-peroxidase detection system (UltraTek HRP Anti- Polyvalent Lab Pack, ScyTek, Logan, Utah, USA) according to the manufacturer’s instructions^29^.

### Statistical analysis

Statistical analysis was performed using GraphPad Prism 5 (GraphPad Software Inc, San Diego California USA). Children were divided in two age groups: young (≤10 years) and old (>10 years). Categorical variables were analyzed using Fisher’s exact test or Chi Square test when necessary. The Kolmogorov–Smirnov test was used for testing normality. T-test or Mann–Whitney test was used to compare the means, when appropriate. Correlations between data were determined using Spearman or Pearson rank correlation index, when appropriate. All tests were two-tailed, and p < 0.05 was considered statistically significant.

To explore the structure of association among variables of tissue microenvironment, EBV presence and age-group, a hierarchical cluster analysis using average linkage and binary simple matching measure with Statistical Package for the Social Sciences 13.0 (SPSS) was performed.

## Supplementary information

Supplementary Tables
