# Supplementary material for: M1-like macrophage polarization prevails in young children with classic Hodgkin Lymphoma from Argentina
Source: Sci Rep. 2019 Sep 3;9:12687. doi: 10.1038/s41598-019-49015-1 (PMC6722052; doi:10.1038/s41598-019-49015-1)
Supplement: Supplementary file 1 — Supplementary Tables [file 41598_2019_49015_MOESM1_ESM.doc]

**M1-like macrophage polarization prevails in young children with classic Hodgkin Lymphoma from Argentina.**

Jimenez O^1^, Barros MH^2^, De Matteo E^1^, Garcia Lombardi M^3^, Preciado MV^1^, Niedobitek G^2^, Chabay P^1^.

^1^Multidisciplinary Institute for Investigation in Pediatric Pathologies (IMIPP), CONICET-GCBA. Molecular Biology Laboratory, Pathology Division, Ricardo Gutiérrez Children’s Hospital, Buenos Aires, Argentina.

^2^Institute for Pathology, Unfallkrankenhaus Berlin, Berlin, Germany.

^3^Oncology Division, Ricardo Gutiérrez Children’s Hospital, Buenos Aires, Argentina.

Table S1: General characteristic of included patients.

|  | **AGE GROUP** | |  | **EBV-STATUS** | |  |
| --- | --- | --- | --- | --- | --- | --- |
| **VARIABLES** | **<10 YEARS** | **>10 YEARS** | p^a^ | **EBV+** | **EBV-** | p |
| **Age** | 31 (67.4%) | 15 (32.6%) | - | 3 – 16 (8)^b^ | 7 – 15 (11)^b^ | 0.006^c^ |
| **Gender**  **Male**  **Female** | 25 (86.2%)  4 (13.8%) | 10 (58.8%)  7 (41.2%) | 0.0126 | 30 (85.7%)  5 (14.3%) | 5 (45.5%)  6 (54.5%) | 0.0126^a^ |
| **Histological Subtype**  **Nodular sclerosis**  **Mixed cellularity**  **Others (LR and LD)** | 3 (11.1%)  22 (81.5%)  2 (7.4%) | 11 (57.9%)  5 (26.3%)  3 (15.8%) | 0.0004 | 7 (20%)  25 (71.4%)  3 (8.6%) | 7 (63.6%)  2 (18.2%)  2 (18.2%) | 0.0085 ^a^ |

EBV: Epstein-Barr virus. ^a^ p-value originated from Fisher´s exact test. ^b^ Minimum/maximum variation; the numbers in brackets represent the mean. ^c^ p-value originated from Mann-Whitney test. When it is not specified, the numbers represent number of cases

Table S2: Correlation coefficients among the evaluated cell subtypes.

| **CELL SUBTYPE** |  | **CELL SUBTYPE** | | | | | | | |
| --- | --- | --- | --- | --- | --- | --- | --- | --- | --- |
|  |  | **CD4+** | **FOXP3+** | **CD8+** | **GrB+** | **CD68+pSTAT1+** | **CD68+CMAF+** | **CD163+PSTAT1+** | **CD163+CMAF+** |
| **CD4+** |  |  | r= 0.08  p= 0.55 | r= 0.03  p= 0.81 | r= 0.4  p= 0.005 | r= 0.29  p= 0.067 | r= 0.22  p= 0.14 | r= 0.016  p= 0.93 | r= 0.16  p= 0.31 |
| **FOXP3+** |  | r= 0.08  p= 0.55 |  | r= 0.11  p= 0.43 | r= 0.30  p= 0.037 | r= 0.32  p= 0.043 | r= -0.1  p= 0.51 | r= 0.21  p= 0.17 | r= -0.12  p= 0.47 |
| **CD8+** |  | r= 0.36  p= 0.81 | r= 0.11  p= 0.43 |  | r= -0.02  p= 0.99 | r= 0.11  p= 0.48 | r= -0.49  p= 0.001 | r= 0.2  p= 0.89 | r= -0.32  p= 0.049 |
| **GrB+** |  | r= 0.40  p= 0.05 | r= 0.3  p= 0.037 | r= -0.002  p= 0.99 |  | r= 0.46  p= 0.002 | r= 0.006  p= 0.96 | r= 0.26  p= 0.097 | r= 0.14  p= 0.38 |
| **CD68+pSTAT1+** |  | r= 0.29  p= 0.067 | r=0.32  p= 0.043 | r= 0.11  p= 0.48 | r= 0.46  p= 0.002 |  | r= -0.14  p= 0.38 | r= 0.56  p< 0.0005 | r= 0.1  p= 0.57 |
| **CD68+CMAF+** |  | r= 0.22  p= 0.14 | r= -0.1  p= 0.51 | r= -0.49  p= 0.001 | r= 0.006  p= 0.96 | r= -0.14  p= 0.38 |  | r= 0.43  p= 0.79 | r= 0.59  p< 0.0005 |
| **CD163+pSTAT1+** |  | r= 0.016  p= 0.92 | r= 0.21  p= 0.17 | r= 0.02  p= 0.89 | r= 0.26  p= 0.097 | r= 0.56  p< 0.0005 | r= 0.04  p= 0.79 |  | r= 0.07  p= 0.69 |
| **CD163+CMAF+** |  | r= 0.16  p= 0.31 | r= -0.12  p= 0.47 | r= -0.32  p= 0.049 | r= 0.14  p= 0.38 |  | r= 0.59  p< 0.0005 | r= 0.07  p= 0.69 |  |

r-value (correlation coefficient) and p-value originated from Spearman´s correlation.

Table S3: Correlations of the evaluated ratios with age group and Epstein-Barr virus (EBV) status.

|  | **AGE GROUP** | |  | **EBV-STATUS** | |  |
| --- | --- | --- | --- | --- | --- | --- |
| **RATIOS** | **<10 YEARS** | **>10 YEARS** | **p** | **EBV+** | **EBV-** | **p** |
| **FOXP3+ : GrB+ cells**  FOXP3 > GrB  GrB > FOXP3 | 19 (61.3%)  12 (38.7%) | 11 (73.3%)  4 (26.7%) | 0.52 | 23 (65.7%)  12 (34.3%) | 7 (63.6%)  4 (36.4%) | 1 |
| **CD68+pSTAT1+ : CD68+CMAF+ cells**  M1 > M2  M2 > M1  M1 ≈ M2 | 24 (85.7%)  1 (3.6%)  3 (10.7%) | 6 (54.5%)  5 (45.5%)  0 | 0.006 | 26 (81.3%)  4 (12.5%)  2 (6.3%) | 4 (57.1%)  2 (28.6%)  1 (14.3%) | 0.21 |
| **CD163+pSTAT1+ : CD163+CMAF+ cells**  M1 > M2  M2 > M1  M1 ≈ M2 | 15 (75%)  2 (10%)  3 (15%) | 3 (23.1%)  9 (69.2%)  1 (7.7%) | 0.0015 | 16 (66.7%)  5 (20.8%)  3 (12.5%) | 2 (22.2%)  6 (66.7%)  1 (11.1%) | 0.032 |

p-value originated from Fisher´s exact test. M1 > M2 or M2 > M1 was defined as one cell population 1.5x higher than the other.
